# Retraction: *Meloidogyne enterolobii* risk to agriculture, its present status and future prospective for management

**DOI:** 10.3389/fpls.2025.1689345

**Published:** 2025-09-01

**Authors:** Frontiers Editorial Office

The journal retracts the 2023 article cited above.

Following concerns regarding the originality of the article, an investigation was conducted in accordance with Frontiers’ policies. It was found that the complaint was valid and that the article should be retracted because of an unacceptable level of similarity, lack of proper attribution to and minimal new information when compared to an article published by Philbrick AN, Adhikari TB, Louws FJ and Gorny AM (2020) *Meloidogyne enterolobii*, a Major Threat to Tomato Production: Current Status and Future Prospects for Its Management. *Front. Plant Sci.* 11:606395. doi: 10.3389/fpls.2020.606395.

This retraction was approved by the Chief Editors of *Frontiers in Plant Science* and the Chief Executive Editor of Frontiers. The authors do not agree to this retraction.

Frontiers would like to thank the concerned reader who contacted us regarding the published article.

